# The effect of financial incentives on patients’ motivation for treatment: results of “Money for Medication,” a randomised controlled trial

**DOI:** 10.1186/s12888-018-1730-y

**Published:** 2018-05-24

**Authors:** Ernst L. Noordraven, André I. Wierdsma, Peter Blanken, Anthony F. T. Bloemendaal, Cornelis L. Mulder

**Affiliations:** 1 Dual Diagnosis Center Palier, Parnassia Psychiatric Institute, 2552 KS The Hague, The Netherlands; 2 Epidemiological and Social Psychiatric Research Institute, Department of Psychiatry, Erasmus University Medical Center, 3015 CE Rotterdam, The Netherlands; 3 Bavo-Europoort Mental Health Care, 3066 TA Rotterdam, The Netherlands; 4 Parnassia Addiction Research Centre (PARC), Brijder Addiction Center, Parnassia Psychiatric Institute, 2553 RJ The Hague, The Netherlands

**Keywords:** Adherence, Motivation, Antipsychotic depot medication, Schizophrenia, Financial incentives

## Abstract

**Background:**

Offering financial incentives is an effective intervention for improving adherence in patients taking antipsychotic depot medication. We assessed whether patients’ motivation for treatment might be reduced after receiving financial rewards.

**Methods:**

This study was part of Money for Medication, a multicentre, open-label, randomised controlled trial, which demonstrated the positive effects of financial incentives on antipsychotic depot compliance. Three mental healthcare institutions in Dutch secondary psychiatric care services participated. Eligible patients were aged 18–65 years, had been diagnosed with schizophrenia or another psychotic disorder, had been prescribed antipsychotic depot medication or had an indication to start using depot medication, and were participating in outpatient treatment. For 12 months, patients were randomly assigned either to treatment as usual (control group) or to treatment as usual plus a financial reward for each depot of medication received (€30 per month if fully compliant; intervention group). They were followed up for 6 months, during which time no monetary rewards were offered for taking antipsychotic medication. To assess treatment motivation after 0, 12 and 18 months, interviews were conducted using a supplement to the Health of the Nation Outcome Scales (HoNOS) and the Treatment Entry Questionnaire (TEQ).

**Results:**

Patients were randomly assigned to the intervention (*n* = 84) or the control group (*n* = 85). After 12 months, HoNOS motivation scores were available for 131 patients (78%). Ninety-one percent of the patients had no or mild motivational problems for overall treatment; over time, there were no significant differences between the intervention and control groups. TEQ data was available for a subgroup of patients (*n* = 61), and showed no significant differences over time between the intervention and control groups for external motivation (β = 0.37 95% CI: -2.49 – 3.23, *p* = 0.799); introjected motivation (β = − 2.39 95% CI: -6.22 – 1.44, *p* = 0.222); and identified motivation (β = − 0.91 95% CI: -4.42 – 2.61, *p* = 0.613). After the 6-month follow-up period, results for the HoNOS and TEQ scores remained comparable.

**Conclusions:**

Offering financial incentives for taking antipsychotic depot medication does not reduce patients’ motivation for treatment.

**Trial registration:**

Netherlands Trial registration, number NTR2350.

## Background

Non-adherence to antipsychotic medication remains a considerable problem in the treatment of patients with psychotic disorders [1, 2]; it is associated with poor clinical outcomes such as increased psychiatric symptoms, hospital admissions, violent crimes and suicide rates [3–5]. Randomised controlled studies demonstrated that medication adherence improved when financial incentives were offered [6, 7]. However, a systematic review by Deci and colleagues (1999) found that people who received performance-contingent rewards showed lower levels of intrinsic motivation than people who received no rewards [8]. This can arise if incentives are perceived as controlling [9]. Furthermore, this negative relationship between external rewards and intrinsic motivation seems to be present both during, and after incentives are being offered [10]. Therefore, it is conceivable that offering financial incentives reduces patients’ motivation, as they may stop their medication intake when incentives are no longer offered. This could negatively affect the long-term treatment, particularly of patients with schizophrenia.

Motivation for treatment, however, is a multidimensional concept. According to the Self-Determination Theory (SDT [11]), motivation to engage in activities ranges from “activities that are completely initiated and controlled by external social forces (such as financial incentives), to activities that are fully self-determined.” Within this continuum, SDT defines three types of motivation [12]. *External* motivation refers to individuals who seek treatment or help due to social pressure or in order to avoid punishment or achieve external rewards (e.g., monetary rewards). *Introjected* motivation states that internal or personal conflicts (e.g., feelings of guilt, shame or anxiety) are the primary reason for remaining in treatment. Finally, *identified* motivation refers to individuals who personally identify with the goals of therapy – who, rather than being motivated by qualifying for rewards or avoiding internal conflicts, seek treatment for themselves. Here, we consider identified motivation as the most self-determined form of motivation, and view this subtype as intrinsic motivation [13].

The aim of this study was to explore, in the context of Money for Medication (M4M), a randomised controlled trial [6], whether offering patients financial incentives to take antipsychotic depot medication reduced their motivation for treatment. Motivation was assessed during a 12-month intervention and a 6-month follow-up period. We also explored the role of clinical variables that have an impact on treatment motivation, such as illness insight [14], medication adherence and the side-effects of antipsychotic medication.

## Methods

### Study design and patients

Between May 2010 and October 2014, a total of 169 patients participated in our M4M randomised controlled trial; a detailed account of the study design has been published in the main trial paper [6]. Patients were recruited from three mental healthcare institutions in the Netherlands: Dual Diagnosis Center Palier, Parnassia, and BavoEuropoort. These organisations primarily treat patients with psychotic disorders and other severe mental illnesses (often with comorbid substance use). Eligible patients were aged 18–65 years, had a psychotic disorder classified by the DSM-IV, had been prescribed or had an indication to start antipsychotic depot medication, were participating in outpatient treatment, and had given written informed consent. Patients were excluded if they were unable to participate due to cognitive impairments or had insufficient understanding of the Dutch language. Before participating in this study, all patients provided written informed consent. The study was approved by the Dutch Medical Ethical Trial Committee of Erasmus University Medical Center (registration number NL31406.097.10), and was registered in the Netherlands Trial Registration (NTR2350).

### Procedure

Patients were selected from the caseloads of the participating treatment teams on the basis of the selection criteria, and were informed about the study by their clinicians. Patients who participated were interviewed at baseline, and after 12 and 18 months. They received €20 remuneration for each completed interview. After the baseline interview, they were randomly assigned to 12 months, either of treatment as usual, or of treatment as usual plus financial incentives to take the antipsychotic depot medication. Randomisation was stratified by treatment site and three potential prognostic factors: sex, comorbid substance-use disorder (absent vs. present), and compliance with antipsychotic medication in the 4 months before baseline (< 50% vs. ≥50%). The principal investigator had no influence on the enrolment process. Patients, clinicians, interviewers, and research assistants were masked to group allocation before, but not after, assignment.

### Treatment as usual and intervention

Patients were randomly assigned to the intervention (*n* = 84) or the control group (*n* = 85). The control group received treatment as usual (TAU), both during the 12-month intervention period and during the 6-month follow-up phase. This treatment was provided by community mental health teams. During TAU, clinicians encouraged patients to take their antipsychotic depot medication as prescribed. If necessary, crisis services were used, or patients were admitted to hospital. All patients received their depot medication at the outpatient clinic, where it was administered by psychiatric nurses.

Patients in the intervention group received TAU, plus a financial reward for every depot of antipsychotic medication they took during the 12-month intervention period. The maximum reward was €30 per month. The amount per taken depot varied according to the frequency of the prescription, which ranged between one and four times per month (i.e., between €7.50 and €30 per depot). After the intervention period, all patients entered the 6-month follow-up period and received TAU without financial incentives.

### Outcomes

To assess patients’ overall motivation for treatment, we used a supplement to the Dutch translation of the Health of the Nation Outcome Scales (HoNOS) [15, 16]. During this structured interview, one item specifically measured motivation (supplement B; “How motivated are you for your current treatment?”), which was rated on a 5-point scale ranging from 0 (no problems) to 4 (very severe problems). On the basis of the skewed response distributions, treatment motivation scores were dichotomised into “no or mild problems” (scores 0, 1 and 2) and “severe problems” (scores 3 and 4). During the course of the study many patients were lost-to-follow up, as they did not show up for appointments with the interviewers. After 12 months, HoNOS motivation scores were available for 131 patients (78%: 66 intervention vs. 65 control); after 18 months, they were available for 109 patients (64%: 60 intervention vs. 49 control).

We also assessed treatment motivation using the Dutch version of the Treatment Entry Questionnaire (TEQ) [12, 17, 18]. This questionnaire consists of 27 items and distinguishes three subtypes of motivation: (1) external, (2) introjected, and (3) identified. External motivation included 12 items (e.g., “*The reason I am in treatment is because other people have pressured me to be here*”); introjected motivation included 6 items (e.g., “*I plan to go through with treatment, because I will feel ashamed of myself if I don’t*”); and identified motivation included 9 items (e.g., “*I decided to follow treatment because it feels important to me to personally deal with my problems*”). Each item was rated on a scale from 1 (strongly disagree) to 7 (strongly agree). Subscale scores were computed by summing the item scores, with higher scores reflecting a higher level of external, introjected or intrinsic motivation. The TEQ was added after about half of the patients were already interviewed at baseline. Therefore, the TEQ was administered to 85 patients at baseline (42 intervention and 43 controls). After 12 months, TEQ scores were available for 61 patients (72%: 27 intervention vs. 34 control); after 18 months, they were available for 49 patients (58%: 21 intervention vs. 28 control).

### Motivation covariates

To explore factors influencing treatment motivation, we analysed the effects of illness insight, side-effects, and medication adherence. To measure patients’ level of illness insight at baseline, we used the Dutch version of the Positive and Negative Syndrome Scale (PANSS) [19]. For item A12 of the PANSS (“Do you have a psychiatric disorder or mental health problem?”) patients were asked to elaborate their answers. Responses were scored on a scale from 1 (illness insight present) to 7 (active denial of having a psychiatric disorder). To monitor common side-effects associated with the use of antipsychotic medication, we also used the 17-item Antipsychotic Side-effect Checklist (ASC) [20]. Each item was rated as “symptom present” or “symptom absent”, and the total number of reported side-effects was calculated (range: 0–17). Finally, we measured medication adherence, which was defined as the Medication Possession Ratio (MPR; [21]), i.e., the number of depots of antipsychotic medication received, divided by the total number of depots of antipsychotic medication prescribed during the 12-month intervention and 6-month follow-up period.

### Statistical analyses

HoNOS motivation scores were dichotomised into “no or mild problems” or “severe problems” at baseline, and after 12 and 18 months. Baseline differences, response rates and motivation trajectories were analysed using (multivariate) logistic and multinomial regression. Sensitivity analyses were conducted using different cut-off scores and trajectory classifications to explore the effect of the dichotomization and combination of HoNOS scores. For TEQ motivation scores, we used generalised linear models with a gamma distribution and logit link to analyse differences between treatment groups. Regression models were compared on the basis of the log-likelihood ratio. In our adjusted models we entered stratification variables as covariates. As the participating mental healthcare teams were reorganised during the study, treatment site was not included. In TEQ motivation models we added baseline values, illness insight, medication side-effects and medication adherence as cofactors. Extended reports on sensitivity analyses and modelling results are available on request from the corresponding author. All statistical analyses were performed using SPSS (version 21.0).

## Results

Table 1 presents the sociodemographic and clinical characteristics of all patients at baseline (*n* = 169).

**Table 1:** Patient characteristics and clinical status at baseline

Variable	Total (*n* = 169)	Intervention group (*n* = 84)	Control group (*n* = 85)
Age mean (SD), years	40.7 (9.8)	40.6 (9.4)	40.7 (10.2)
Gender, *N* (%)			
- Male	127 (75.1)	61 (72.6)	66 (77.6)
Patients > 50% medication adherence, *N* (%)	135 (79.9)	68 (80.0)	67 (79.8)
Place of treatment, *N* (%)			
- The Hague	46 (27.2)	18 (21.4)	18 (21.2)
- Rotterdam	123 (72.8)	66 (78.6)	67 (78.8)
Substance use disorder, *N* (%)	94 (55.6)	48 (57.1)	46 (54.1)
Antipsychotic Medication side effects, mean (SD)	4,8 (4,0)	5,3 (4,0)	4,3 (3,9)
Illness insight; median (interquartile range) (range 1–7)	3 (1–4)	3 (1–4)	2 (1–4)
Health of the Nation Outcome Scales (HoNOS), *N* (%)			
- No motivational problems	136 (80.4)	66 (78.6)	70 (82.3)
- Severe motivational problems	28 (16.6)	17 (20.2)	11 (12.9)
- Item missing	5 (3.0)	1 (1.2)	4 (4.8)
Treatment Entry Questionnaire, (TEQ) *N* (%)	85 (50.3)	42 (50.0)	43 (50.6)
- External motivation; mean (SD), (range 12–84)	18.4 (7.8)	17.6 (8.1)	19.0 (7.6)
- Introjected motivation; mean (SD), (range 6–42)	18.7 (10.6)	19.3 (9.4)	18.2 (9.4)
- Identified motivation; mean (SD), (range 9–63)	28.5 (10.0)	28.6 (10.9)	28.5 (9.2)

### Trajectories of motivation (baseline – 12 months)

Four categories were distinguished: (1) patients with mild or no problems at baseline and after 12 months (*n* = 106; 81%); (2) patients with severe motivational problems at baseline, who showed an improved treatment motivation after 12 months (*n* = 13; 10%); (3) patients with severe motivational problems at baseline, who did not improve (*n* = 8; 6%); and (4) patients with mild or no problems at baseline who showed severe motivational problems after 12 months (*n* = 4; 3%). Patients with HoNOS scores at baseline and after 12 months (*n* = 131) were compared with patients who had only HoNOS baseline scores (*n* = 35). Logistic regression analyses were performed with patient status (i.e., being in the subgroup or not) as dependent variable and with patient characteristics as predictor variables (i.e., age, gender, substance-use disorder, medication adherence, ethnicity, income, and illness insight). There were no significant differences between the HoNOS subgroups (not reported here to save space).

Between trajectories there were no differences for the intervention and control patients (β = − 0.412, 95% CI -1.15-0.33, *p* = 0.274; reference category 1). Sensitivity analysis yielded similar results.

### Main effects on motivation subtypes

We compared patients with TEQ scores at baseline and after 12 months (*n* = 61) with those who had only a TEQ baseline measure (*n* = 24). This showed a significant difference for baseline medication adherence (β = 0.35, 95% CI 0.01–0.06, *p* = 0.008). The TEQ subgroup (n = 61) also showed a difference with the rest of the sample (*n* = 108) for baseline medication adherence (β = 0.03 95% CI: 0.02–0.05, *p* = 0.000) and substance use (β = 1.24 95% CI: 0.48–1.99, *p* = 0.001).

Adjusted regression models consisted of the stratification variables (i.e., condition, gender, substance use, and baseline medication adherence), and baseline motivation. After 12 months of offering financial incentives, we found no effects of treatment condition on any type of motivation assessed on the TEQ. There were no mean score differences in external motivation between the intervention group (19.4 [SD:7.0]) and control group (20.4 [SD:6.4] (adjusted difference of 0.37 points (95% CI -2.5–3.2, *p* = 0.799)). Similarly, the mean score difference in introjected motivation was non-significant between the intervention group (18.4 [SD:9.4]) and control group (21.0 [SD:10.1] (adjusted difference of − 2.4 points (95% CI -6.2–1.4, *p* = 0.222)). Finally, we found that the mean score for identified motivation was not lower in the intervention group (27.5 [SD:9.8]) than in the control group (27.9 [SD:9.4]) (adjusted difference of − 0,91 points (95% CI -4.4–2.61; *p* = 0.613)).

### Motivation covariates

To explore the association with motivation, we added the following to the model: medication side-effects, illness insight (assessed at baseline), and medication adherence (Table 2). Only illness insight had significant main effects on introjected motivation (β = − 1,33 95% CI: -2,47 – -0,19, *p* = 0,023) and identified motivation (β = − 1,65 95% CI: -2,72 – -0,59, p = 0,002). This suggests that less illness insight (reflected by higher scores) is associated with less introjected motivation for treatment, and less identified motivation for treatment. There was no interaction effect of illness insight and condition on either introjected motivation (β = 0,49 95% CI: -1,80–2,78, p = 0,675) or identified motivation (β = 0,63 95% CI: -1,43–2,69, p = 0,548).

**Table 2:** Coefficients for regression model, adjusted for medication side-effects, illness insight, or medication adherence

	External motivation			Introjected motivation			Identified motivation		
	β	95% CI	*p* value	β	95% CI	*p* value	β	95% CI	*p* value
Intercept	12.45	2.96; 21.94	0.011	5.71	−6.01; 17.41	0.341	7.41	−3.85; 18.67	0.197
Condition	0.37	−2.49; 3.23	0.799	− 2.39	−6.22; 1.44	0.222	−0.91	−4.42; 2.61	0.613
Gender	1.61	−1.82; 5.04	0.358	0.57	−4.09; 5.24	0.811	−3.76	−8.04; 0.52	0.085
Substance use	1.09	−1.88; 4.06	0.472	0.77	−3.26; 4.79	0.708	−0.28	−3.97; 3.42	0.884
Medication adherence baseline^a^	−0.03	−0.11; 0.06	0.548	0.04	−0.07; 0.16	0.506	0.09	−0.02; 0.19	0.103
Motivation baseline^b^	0.43	0.25; 0.61	< 0.001	0.61	0.41; 0.81	< 0.001	0.56	0.39; 0.72	< 0.001
Medication side-effects	−0.12	−0.54; 0.29	0.571	−0.11	−0.67; 0.46	0.714	0.34	−0.16; 0.84	0.181
Illness insight	−0.29	−1.13; 0.54	0.486	−1.33	−2.47; − 0.19	0.023	−1.65	−2.72; − 0.59	0.002
Medication adherence 12 months	−0.03	− 0.14; 0.07	0.542	− 0.02	−0.16; 0.12	0.796	0.02	−0.11; 0.15	0.711

^a^ Medication adherence in the 4 months prior to baseline ^b^Motivation measured at baseline for each type of motivation

### Follow-up period (baseline – 18 months)

After the 6-month follow-up period, HoNOS motivation supplement scores were available for 109 patients (64%). These were divided into four categories: (1) patients who continued (during 12–18 month follow-up) to have mild or no motivational problems for treatment (*n* = 81; 75%); (2) patients who had previously had severe motivational problems (at baseline), but had now improved (*n* = 9; 8%); (3) patients who continued to have severe motivational problems throughout the study (from 0 to 18 months, n = 8; 7%); and (4) patients who had had mild or no motivational problems before but had severe problems at follow-up (*n* = 11; 10%). Per category, there were no differences between the number of intervention and control patients.

The adjusted model for 18-month TEQ scores showed no significant differences between the intervention and control groups for external motivation (β = 0.89 95% CI: -4.68 – -2.89, *p* = 0.644), introjected motivation (β = − 1.47 95% CI: -5.77 – -2.83, *p* = 0.449), and identified motivation (β = − 2.15 95% CI: -6.14 – -1.84, *p* = 0.291). There was a significant main effect for illness insight on identified motivation (β = − 1.24 95% CI: -2.44 – -0.03, *p* = 0.044), but no interaction effect of illness insight and treatment condition.

## Discussion

This study was intended to establish whether offering patients financial incentives to take antipsychotic depot medication would reduce their motivation for treatment. Our findings suggest that it did not. Over time, patients who received financial incentives did not differ with respect to various types of motivation from those who received treatment as usual. In addition, after the discontinuation of financial incentives, their medication adherence remained significantly higher.

After 12 months, 91% of the patients showed no or only mild motivational problems. During the 6-month follow-up period, when financial incentives were no longer offered, a majority of the patients (83%) were still motivated for treatment, whereas relatively few (17%) reported having little motivation for or resistance to their current treatment. In sum, this study indicates that offering and then discontinuing financial incentives to patients with psychotic disorders does not reduce their motivation for clinical treatment. It is particularly noteworthy that intrinsic motivation for treatment (which refers to individuals who identify personally with the goals of therapy) was not lower in patients who received financial incentives than in control patients. Similarly, patients’ external motivation for treatment was not higher after they had received financial incentives.

### Strengths and limitations

This is the first study to assess the impact of financial incentives on patients’ motivation for treatment. Using two questionnaires, HoNOS addendum and TEQ, to assess treatment motivation, we found that offering financial incentives had produced no negative consequences. The TEQ enabled us to measure motivationand also to distinguish three subtypes of motivation.

However, the first limitation is that loss to follow-up was considerable for the motivational outcome measures in this study as patients often did not show up for scheduled appointments with the interviewers. Organisational factors prevented us from administering the TEQ to more than only a subgroup of patients and selection bias is likely to be an issue. The higher levels of medication adherence and fewer diagnoses of substance-use disorder in this subgroup showed that they performed somewhat better at the start of treatment than the rest of the sample did. These patients may therefore have been more motivated throughout the study: for example, they may have had a high intrinsic motivation for treatment. As there was a danger that financial incentives would make them susceptible to adhering to treatment more for the external rewards than for themselves, it is important to note that there was no change in their intrinsic motivation when the financial incentives ended. For other patient characteristics, this subgroup did not differ significantly from the rest of the sample.

The second limitation is that overall treatment motivation was assessed on the basis of one item from the HoNOS-addendum scale, which thus reduced psychometric validity.

Another limitation is that, when the incentives ended, external motivation for treatment did not differ between the intervention group and the control group. It might be argued that the financial incentives may not have been great enough to cause major changes in patients’ external motivation for treatment. However, they were sufficient to improve patients’ medication adherence during the intervention period [6], and medication adherence remained significantly higher for the intervention group when financial incentives were no longer offered.

### Further implications

Even though types of motivation did not differ between the intervention and control groups, there was a significant main effect for illness insight. Poor illness insight at study entrance appears to have been associated with less introjected and intrinsic motivation for treatment. These effect sizes were rather small, however. Also, when the intervention had finished, there seemed to be no so-called “crowding out” effect [22]. In other words, not only had patients not become externally motivated when the incentives were removed, they had not lost their intrinsic motivation.

## Conclusions

Financial incentives improve adherence to antipsychotic depot medication in patients with psychotic disorders. The current study suggests that offering such incentives does not reduce patients’ motivation for clinical treatment. It is particularly relevant that patients who received financial incentives had neither lower intrinsic motivation for treatment nor higher external motivation. These results remained similar during the follow-up period, when incentives were no longer offered. Financial incentives can therefore be seen as an effective and relatively safe intervention for improving depot-medication adherence among patients with psychotic disorders.

## Abbreviations

ASC: Antipsychotic Side-effect Checklist
HONOS: Health of the Nation Outcome Scales
M4M: Money for Medication
MPR: Medication Possession Ratio
PANSS: Positive and Negative Syndrome Scale
SDT: Self-determination theory
TAU: Treatment as usual
TEQ: Treatment Entry Questionnaire
